# Media perception and trust among disaster survivors: Tsunami survivors' interaction with journalists, media exposure, and associations with trust in media and authorities

**DOI:** 10.3389/fpubh.2022.943444

**Published:** 2022-08-02

**Authors:** Liselotte Englund, Kerstin Bergh Johannesson, Filip K. Arnberg

**Affiliations:** ^1^Department of Risk and Environmental Studies, Faculty of Arts and Social Sciences, Karlstad University, Karlstad, Sweden; ^2^Department of Medical Sciences, National Centre for Disaster Psychiatry, Uppsala University, Uppsala, Sweden

**Keywords:** authorities, disaster communication, journalists, media ethics, media exposure, natural disaster, survivors experiences, trust

## Abstract

A critical part of disaster communication is media coverage in the interface of the afflicted, media, and authorities. One communication key is building trust. Disaster survivors encounter journalists in a high-stress context, but little is known about their perceptions of these interactions and the subsequent media exposure. The aim of this study is to explore how survivors 6 years after a major disaster perceived their encounters with journalists and exposure in the media, as well as their level of trust in the media, compared with government and authorities. Data were used from a longitudinal study of Swedish tourists, repatriated from the 2004 Indian Ocean tsunami, surveyed up to 6 years after the tsunami to assess posttraumatic stress (PTS) and effects on mental health. At 6 years after, the survey included questions about survivors' perceptions of journalist interactions (reported by *n* = 311), of their own media exposure (*n* = 177), and survivors' trust in media organizations and public authorities (*n* = 1,181). Tsunami survivors mainly perceived interactions with journalists as being professional. There were 14% who reported that the interactions were supportive and 17% that the interactions were a strain. Similarly, most participants had a neutral view concerning the subsequent media coverage or exposure, although 12% experienced media exposure as stressful and 12% reported that it had been involuntary. Finally, the survivors indicated higher confidence and trust in Swedish radio and TV as compared to the Swedish authorities, and the participants' level of trust in the media was associated with their perceptions of journalists, *r* = 0.34, *p* < 0.001, and media coverage, *r* = 0.47, *p* < 0.001. Disaster survivors mainly agreed with emotionally neutral statements about interacting with the media, the performance of journalists on site, and their own media exposure. Nonetheless, a substantial minority found the encounters and exposure to be negative, and the results suggest a link between personal experiences or perceptions and trust in the media.

## Introduction

Media coverage and trust in the media and authorities are important aspects in cases of communicating a crisis or a disaster. This was not least shown during the COVID-19 pandemic outbreak in 2020. Well-functioning independent media are of the utmost importance from a democratic perspective. A cross-disciplinary research perspective is needed to understand the complex area of communicating a disaster through media, as well as the effects of media exposure on individuals. How media and individual journalists perform on site and how the published outcomes appear may be important factors in building people's trust in disaster communication and crisis management through media, authorities and governments.

Major traumatic events, such as disasters, usually activate intensive media coverage during the acute phase as well as in the long-term recovery phase. Media serve an important function as a channel of information to the public in connection with accidents and disasters. Nonetheless, there are problems in the encounter between the media and the affected victims in situations of vulnerability and grief. Interactions with reporters and photographers can be strongly felt as intrusive by the victims of the disaster (1–5). Complying with media ethics guidelines in relation to victims, survivors, and relatives is especially important in different kinds of traumatic events at the levels of both the individual and society. In addition, the issue of trust in media and authorities may be affected in different ways.

In 2004, the earthquake in the Indian Ocean and the following tsunami devastated coastal regions in Southeast Asia. Between 220,000 and 300,000 people perished (6), and more than 5 million victims were estimated to have become homeless or were forced to resettle. At the time of the tsunami, thousands of tourists from Europe visited South East Asia. Approximately 7,000 Swedish citizens were estimated to have been in the most affected areas, and around 1,500 were injured in the tsunami. As many as 543 Swedes lost their lives, of whom 140 were children. In relation to the population, Sweden was one of the most affected countries in Europe (6). The event led to worldwide and extensive media coverage.

Previous studies have demonstrated that severe disaster exposure, physical injury (7–9) and traumatic bereavement (10) have a considerable impact on psychological distress and appear to slow down the recovery process. The long-term follow-ups with Swedish tsunami survivors have indicated that even if a majority has recovered from mental health problems, 11–16% of survivors with high disaster exposure severity still suffer from post-traumatic stress reactions 6 years after the disaster (11, 12).

Survivors' stress reactions in accidents and disasters have been studied extensively while research on survivors' experience of encounters with the media at the accident site and experience of the subsequent reporting is still rare (1, 3, 13–17) despite the ubiquity of media presence during large-scale incidents.

However, an earlier Swedish study found that most journalists covering the 2004 tsunami disaster acted in a professional manner, in spite of the fact that working conditions were extremely difficult (18). Professional conduct among journalists was apparently in compliance with the Rules of Professional Conduct in the Code of Ethics for Press, Radio and Television in Sweden (19). The Swedish rules states that “…it is important that journalists show due respect when working in the field and that journalists while on duty strive to report correctly, in order to retain the confidence of the general public.” The Rules of Professional Conduct in the Code of Ethics for Press, Radio and Television in Sweden also declares that “trust in the media and its employees is built upon following the rules of professional conduct” (19). Systematic and ethical violations in the media were, according to this Swedish tsunami study, perceived as rare (18).

It is a challenge to media agencies, editorial staff and individual journalists to ensure that media reporting is ethically sound and accurate with reasonable consideration of the individuals who are affected. Negative emotions may be triggered by media coverage that is perceived as intrusive (16). At the same time, disaster survivors often agree to be interviewed. After the July 22, 2011, terrorist attacks in Norway, hospital staff testified that survivors themselves invited the media to their hospital rooms (20). However, shortly after the event, it may be difficult for a survivor to understand the consequences of media participation (1, 2).

A few international studies discuss the reporters' crisis reactions (21–24), but, to date, studies of reactions among the survivors after interacting with reporters are limited, especially concerning the potential effects on survivors' mental health. A study of adolescents' media exposure after a school shooting indicated that being interviewed by the media was associated with increased posttraumatic stress (PTS) reactions (5). Positive and negative experiences with media related to PTS reactions were investigated in face-to-face interviews with survivors of the 2011 Utøya Island terrorist attack in Norway. The majority of survivors perceived their media contact and participation as positive. Media participation was unrelated to PTS reactions. However, survivors who found media participation distressing had more PTS reactions (15). The authors concluded that reporters should take care when interviewing victims, and clinicians should be aware of media exposure as a potential additional strain on victims.

An interview study of surviving passengers at two Swedish train crashes (2) pointed out three major ways survivors perceived the presence of the media at disaster sites, namely as straining, negligible, or, in some cases, supportive. In a later interview study of Swedish survivors from the Estonia ship disaster in 1994 (1), their perceptions were more complex: Survivors described a wide array of experiences from their contacts with the disaster journalists and being exposed in the media. Four categories common for both media contacts and media exposure were extracted from the Estonia study, namely strain, support, rationality, and evasion.

The tsunami disaster in 2004 received more coverage in Swedish media than any other event during the past few decades (25). Initial news reports primarily focused on devastation and death tolls, eyewitness accounts and repatriation. Survivors and injured persons were among the most commonly cited categories in the Swedish press. Others were politicians, officials and people in general (ibid.).

Besides trust in the media, survivors' trust in public authorities plays an important role in the aftermath of a disaster and during crisis events (18, 26–28). Lack of trust, information management and support could become a challenge in a situation where survivors, relatives and citizens are in need of information and support to be able to gain an understanding of a disaster at the same time. If crisis managers do not act and respond as quickly as possible, the need for information will be filled by other actors, such as media (18).

Institutional trust, that is, trust in government and authorities, can develop differently over time among directly affected groups compared to a general population after a traumatic event, although being directly affected does not necessarily imply weakened institutional trust (28). Among disaster victims, low institutional trust is shown to be associated with more mental health problems (29).

However, direct trauma experiences do not necessarily imply weakened institutional trust, according to a Norwegian study (28). In addition, low institutional trust among victims' may impact on their potential healing (29). Trust in media's role in relation to the tsunami disaster was central to receiving knowledge and information about the event according to a study of Swedish citizens' opinions and trust in authorities, media and politicians after the tsunami disaster (30). The study indicated that Swedish citizens considered the government to be ultimately responsible for what they perceived as a poor disaster response. In contrast, the media managed to maintain its position as trustworthy.

Every crisis or disaster situation comes with new challenges, both for the media and for authorities handling the crisis. In the general guidelines of the Board of Swedish Health and Welfare, the importance of conducting rescue operations in a humane and dignified way is emphasized, and this includes providing protection from media exposure as well as timely collaboration with the media (31). Situations such as interviews with survivors trying to handle their stress reactions and with eye-witnesses of accidents and disasters are not covered in the Code of Ethics for Press, Radio, Television in Sweden (19). The Swedish rules of publicity include guidelines for individual integrity, i.e., showing greatest possible consideration for victims of crimes and accidents whether news organizations are publishing names and/or pictures. The rules “serve as protection of the individual against publicity damages, beyond what the legal system can offer” (ibid.). There are also different sets of other national media ethics rules, slightly diverging from the Swedish guidelines. The US-based organization Society of Professional Journalists (SPJ) declares four principles as the foundation of ethical journalism, of which one is “Minimize harm” (32, 33). This can mean showing compassion for those who may be affected by news coverage (33). The term Global Media Ethics, used in some context, is more of a set of principles and standards for the practice of journalism in an age of global news media (34, 35). As Ward (35) notes: “... journalism ethics asks what journalists and news organizations should do, ethically, given their role in society. Journalists have ethical duties to perform and norms to honor because, as human beings, they fall under general ethical principles such as to tell the truth and to minimize harm, and because they have social power to frame the political agenda and influence public opinion. With power comes responsibility” (p. 3–4).

The aim of this study is to explore two main topics, based on the following four questions:

Journalist interaction and media exposure related to mental health:

1. How do survivors perceive, in retrospect, journalists' professional behavior according to the Code of Ethics for Swedish Journalists, regarding (a) encounters with journalists on the disaster site and (b) the survivors' media exposure?
2. Are survivors' retrospective recollections of journalists' conduct related to age or gender, disaster exposure severity, or levels of posttraumatic stress?

Perceptions of trust in media, government and authorities related to media exposure:

3. How does survivors' trust in the media relate to their trust in the government's and authorities' handling of the effects of the tsunami disaster?
4. Do survivors' perception of trust in the media and authorities relate to their perceptions of journalist interactions and media exposure shortly after the disaster?

## Materials and methods

### Procedure

This study used a subset of data from a three-step longitudinal follow-up of Swedish citizens repatriated from the tsunami of South East Asia in 2004. They were registered by Swedish authorities at national airports during the first 3 weeks after the disaster and included individuals at least 16 years of age (*n* = 10,501; 77% of those registered) were then invited to a postal survey after 14 months (T1) (12). The respondents from that survey (*n* = 4,910) were then asked to participate in surveys 3 years (T2) (7) and 6 years after the disaster (T3) (11). The surveys included questions about the participants' demographics, disaster exposure and bereavement, about their lives before and after the disaster (e.g., adverse events in childhood, adulthood and post-tsunami; use of health services; social support).

The present study includes a subset of the 2,643 participants who responded to the 6-year follow-up survey and answered questions about their interaction with journalists (reporter or photographer) and journalists' performance on site in Southeast Asia shortly after the disaster. The data collection was conducted by the National Centre for Disaster Psychiatry, Uppsala, Sweden, in collaboration with the Centre for Family and Community Medicine, Karolinska Institute, and the Institution for Medical Epidemiology and Biostatistics at the Karolinska Institute, Stockholm. The Regional Ethical Vetting Board in Uppsala, Sweden, approved the study (no. 2005:157, 2010:147).

### Participants

There were 2,612 participants who responded to the question about interacting with a journalist (reporter or photographer) in Southeast Asia shortly after the disaster. There were 311 (13.5%) participants who reported that they had interacted with a journalist and were of primary interest. The 2,301 participants who reported no interaction were used as a comparison sample. Among those who reported interaction with a journalist, 177 (57%) endorsed having recollections of being exposed to media coverage shortly after the disaster.

See Table 1 for sample characteristics. The sample was predominantly female, and the age ranged from 21 to 91 years of age. The participants were mainly living in a relationship, and few participants reported that they were unemployed or on sick-leave at the time of the survey. As for disaster exposure severity, about half of the sample were categorized into the high exposure severity group.

**Table 1:** Sample characteristics.

**Characteristic**	**Interaction with journalist**		**Total**
	**No**	**Yes**	
Age, M (*SD*)	49 (14)	49 (12)	49 (14)
Women, *n* (%)	1,362 (59%)	166 (53%)	1,528 (59%)
Unemployed or on sick-leave, *n* (%)^a^	88 (3.9%)	14 (4.5%)	102 (3.9%)
Educational attainment >12 years, *n* (%)^b^	1037 (45%)	155 (50%)	1,192 (46%)
In relationship, *n* (%)	1,781 (77%)	250 (80%)	2,031 (78%)
**Disaster exposure severity**, ***n*** **(%)**^c^			
High	957 (43%)	211 (69%)	1,168 (46%)
Moderate	736 (33%)	77 (25%)	813 (32%)
Low	532 (24%)	17 (6%)	549 (22%)

*Margin totals are 2,612 unless otherwise stated due to missing data*.

a *N = 2,600*.

b *N = 2,601, data from survey at 1 year after disaster*.

c *N = 2,530, data from survey at 1 year after disaster*.

### Measures and predictors

The survey included standard questions about age, gender, employment status, education, and relationship status. Age was treated as a continuous variable. Gender (men/women) and educational attainment (>12/ ≤ 12 years of education) were treated as binary variables. In Sweden, more than 12 years of education corresponds to having started university studies. Employment status was defined as the main occupation that the participant had at the time of the survey (working full-time, part-time, studying, on parental leave, unemployed, on sick-leave, retired). Data for educational attainment and disaster exposure severity were drawn from the first survey in 2006. The key question for this study was whether or not they had “any interaction with a journalist (reporter or photographer) in direct relation to the tsunami disaster (on site in Southeast Asia).” The participants could respond yes or no. If yes, they were instructed to respond to additional questions about their perceptions about these interactions and, if relevant, any media exposure. They were also asked to briefly state, in free text, where and how the interactions took place.

#### Disaster exposure severity

The severity of disaster exposure was categorized into two direct exposure groups—high and moderate exposure—and one indirect exposure group, based on responses to 30 items in the first survey. The high exposure group included participants who indicated that they had been exposed to life threat, being caught by or had been close to being caught by the tsunami waves. The moderate exposure group included participants who indicated, “No, I was not in the neighborhood of being caught by the tsunami wave,” but who indicated one or more of the following: witnessing corpses, others suffering, or forlorn children; had helped other victims; subjectively felt a threat to life, physical injury to themselves or others, loss of relatives or being worried about the fate of their family/relatives. The indirect exposure group included those participants who were passengers in the same aircrafts repatriated back to Sweden and who could have been indirectly exposed, e.g., by being in close vicinity or by talking to affected persons during transportations.

#### Perceptions of journalist conduct

Participants who indicated that they had interacted with a journalist responded to six questions about whether the journalists acted professionally and ethically. The questions about journalist conduct were formulated based on the Code of Ethics for Press, Radio and Television in Sweden (19, 36) and were also based on previous research (1, 2, 21, 37, 38). These questions asked about whether the presence of media was perceived as a (1) strain or (2) support, (3) if the journalists showed appropriate consideration for the participant or (4) for other survivors, (5) showed respectful conduct, and (6) behaved professionally. It was up to the participants to define for themselves the meanings of “appropriate” and “professionally.” The participants used a five-point scale ranging from “strongly disagree” to “strongly agree” to indicate their level of agreement with each statement. As these items were untested before, a reliability analysis was conducted and is provided in the Appendix. The analysis suggested that the six items worked sufficiently well to be analyzed as a total score. The total score represented the mean score of the items, with scores reversed for the question about strain. The range of the mean score was 0–4 and a higher score represented a more sympathetic view of journalists on site.

#### Perception of being exposed in the media

The survivors who endorsed having been exposed in the media (i.e., all kinds of publicity/media coverage about oneself, for example in written text, press photos, radio, TV or on the web) were asked to respond to eight questions about the media coverage being perceived as (1) straining, (2) supportive, (3) positive, (4) negative, (5) true, (6) relevant, (7) involuntary, and (8) leading to publicity damage. The response options were the same as for the questions about perceptions of journalist conduct. The set of questions also included a ninth item asking whether media served an important role as a channel for information, which was excluded after a reliability analysis of these items conducted due to these items being untested (see Appendix). The analysis suggested that the eight items could be condensed into a total score. The responses were used to compute a mean score of experience of the media exposure, with negative items reversed (items 1, 4, 7, and 8). The responses were summed up to create a total score indicating the overall experience of the participants' perceptions of their media exposure. A higher score represented a more sympathetic view of media exposure.

#### Trust in media and in authorities

A part of the survey explored the survivors' trust in government and authorities and in the media. The participants rated their trust on eight items involving how the government, authorities and the media managed the consequences of the disaster. The survey question was: “What trust do you have in the following institutions' management of the tsunami disaster and its aftermath?.” The items included the (1) government, (2) governmental authorities, (3) regional and (4) local authorities, (5) radio and television, (6) national morning papers, (7) evening papers/tabloids, and (8) regional or local papers. The participants indicated their level of trust for each institution on a response scale from very low trust (1) to very high trust (5), or could choose to abstain from rating their level of trust for a particular item. The questions were drawn from the yearly Swedish SOM Surveys (39), which addresses trust in governmental agencies and the media. These surveys are conducted by the SOM Institute, an independent national survey research center at the University of Gothenburg, Sweden.

#### Posttraumatic stress reactions

The Impact of Event Scale-Revised (IES-R; Weiss, 2004) is a widely used self-rating scale to assess posttraumatic stress and includes eight items of intrusion, eight related to avoidance, and six assessing hyperarousal reactions. The respondents rate how distressing the reactions have been during the past 7 days on a five-point scale ranging from 0 (*not at all*) to 4 (*extremely*), yielding a total score of 0-88. The responses are summed to achieve a total score of the severity of posttraumatic stress reactions. The total score is a valid proxy as compared to a structured clinical interview (40).

### Statistical analysis

The survey responses were screened for valid responses. The distribution of missing values on the primary variables was analyzed. Among those who endorsed journalist interactions there were 34 participants (11% of 311) with 136 incomplete responses (7.3% of all responses) to the six questions about their perceptions of these interactions. There were nine participants (5.1% of 177) with 28 incomplete responses (2.0% of all responses) to the eight questions about media exposure. The missing values were imputed by using the predictive mean matching algorithm. The missing values were predicted by using the other variables in the set of questions together with gender, age, and education as predictors. Total scores were then calculated for each set of questions. For the questions on trust, several respondents mistakenly skipped these items due to unintended ambiguities in the survey instructions: 59% (*n* = 1,365) of those who responded no to interaction with a journalist and 21% (*n* = 66) of those who responded yes had missing items on all of these items (*n* = 1,431). These were not imputed, whereas the responses for 86 participants with a mean of 1.77 (Mdn = 1) missing responses were imputed with the same method as the previous imputations, yielding 1,181 respondents to these items.

The distribution of responses to the questions on journalist interactions, media exposure, and trust were described with standard metrics of central tendency and distribution as well as with a balance score. The balance score is used in the yearly Swedish SOM surveys by the SOM Institute (41). In the present study, the balance score was computed for each item by subtracting the total proportion of participants who endorsed the responses “agree not at all” or “agree to a fairly low degree” from the proportion of participants who responded with fairly high or very much agreement. The balance score thus ranges from −100 to +100, where −100 indicates that all participants show little or no agreement and +100 indicates that all participants show fairly high or very much agreement with an item (42). Bivariate associations were analyzed by using correlations, *t*-tests, univariate ANOVA, and chi-square tests depending on the relevant variables. *P* ≤ 0.05 were considered statistically significant. All analyses were conducted with IBM SPSS Statistics v. 27.0.1.

## Results

### Journalist interactions and media exposure

There were 311 participants who reported having interactions with one or more journalists in relation to the disaster. There were 281 (90%) who provided some form of free-text response to a question about in what context these interactions took place. These responses were of varying quality and did not always contain the requested information. Of those participants who provided the requested information, some noted that they took place at hospitals (*n* = 24), hotels (*n* = 15), or airports (*n* = 22) in the disaster areas. Others took place at various other locations (e.g., consulate, church). Some responses mentioned interviews conducted over the telephone. Most references to the press concerned Swedish media although also some referred to interactions with international media.

We examined associations between the characteristics of the survivors and the probability of endorsing journalist interactions, as it might reflect the representativity of the survivors who spoke to journalists after disasters. Men had slightly higher probability of interacting with journalists (13.4%) as compared to women (10.9%), but the difference was not statistically significant, χ^2^ = 3.82, *p* = 0.057. Similarly, neither age (*t* = 0.31, *p* = 0.75) nor level of education (χ^2^ = 2.47, *p* = 0.13) was related to probability of journalist interactions. Those who were highly exposed in the disaster were more likely to have had interactions with journalists (*n* = 211; 18%) than those who were in the moderate (*n* = 77; 9.5%) or low exposure groups (*n* = 17; 3.1%), χ^2^ = 86.5, *p* < 0.001. Related to the higher exposure level among those who had interactions with a journalist, the level of posttraumatic stress reported at the 1-year survey was higher among those who reported interactions with journalists (IES-R *M* = 23.9) as compared to those who did not (IES-R *M* = 18.2), *t* = 5.42, *p* < 0.001.This difference remained at the 3-year survey (IES-R *M* = 17.3 vs. 12.3, *t* = 5.39, *p* < 0.001) and the 6-year survey (IES-R *M* = 16.4 vs. 11.5, *t* = 5.94, *p* < 0.001).

### Perceptions of interactions with journalists

**Figure 1:**
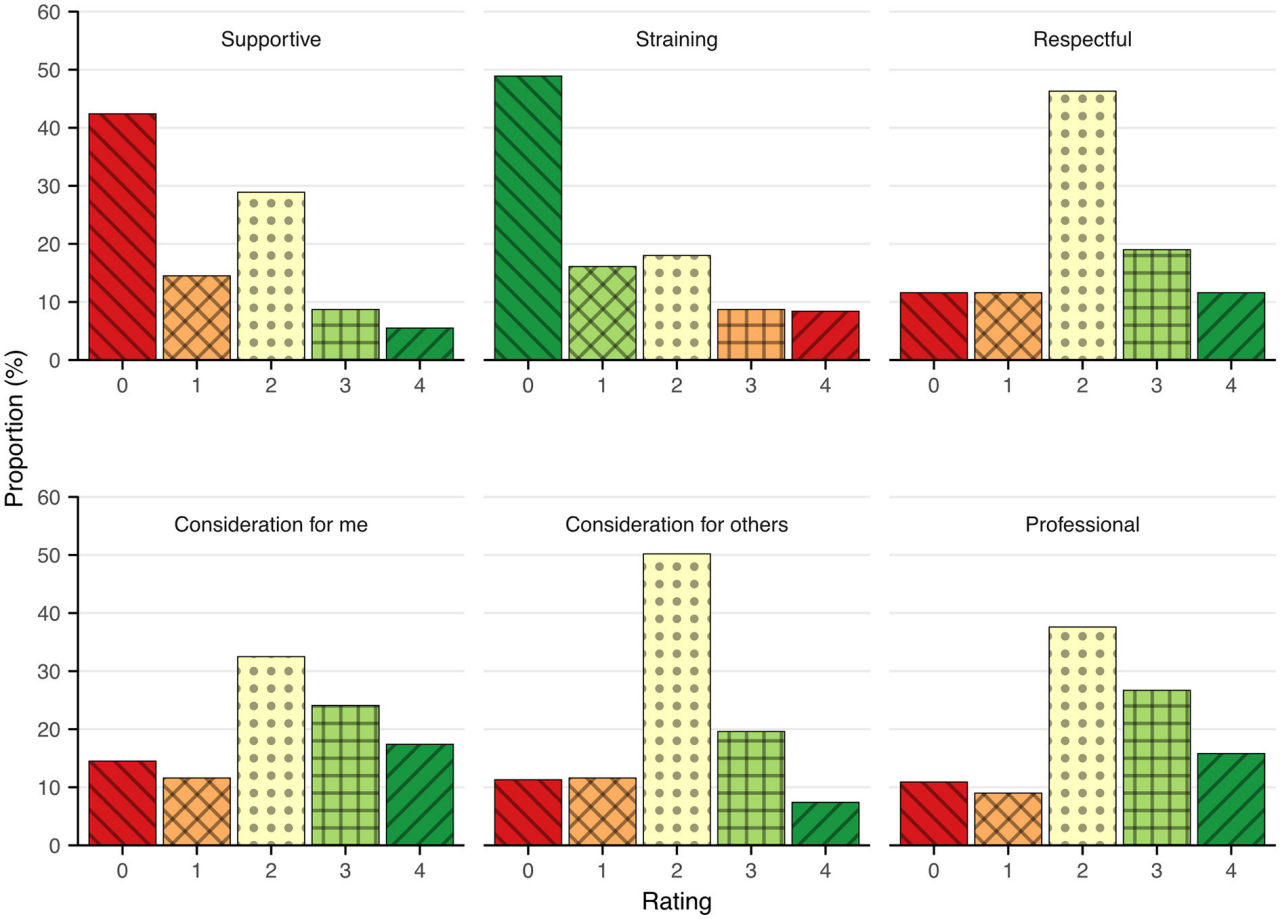
Distribution of Swedish tsunami survivors' perceptions of interactions with journalists after the disaster. Ratings ranges from no agreement (0) to agreeing very much (4).

The retrospective ratings of journalist conduct were quite diverse, and many respondents chose the neutral alternative, neither agreeing nor disagreeing with statements about journalists' conduct. The mean score was 2.1 (*SD* = 0.93), the median was 2, and the range was 0–4. Table 2 summarizes the participants' perceptions of interacting with journalists, and the response distributions are depicted in Figure 1.

**Table 2:** Perceptions of interaction with journalists among 311 tsunami survivors.

	***M***	***SD***	**Balance score**
Strain	1.12	1.33	−47.9
Support	1.20	1.23	−42.8
Consideration for me	2.18	1.27	15.4
Consideration for others	2.00	1.03	4.2
Respect	2.07	1.11	7.4
Professional behavior	2.27	1.16	22.5

*The items range from 0 to 4. The balance score ranges from −100 to +100*.

The distributions of responses are summarized also in the balance score, which indicated that the participants' strongest dissenting opinions were about the items with high emotional valence, that is, the presence of journalists as being a strain or a support. Approximately one fourth (26%; *n* = 81) disagreed with the statement that the journalists showed fair consideration toward themselves or toward others. Among the participants, 17 percent (*n* = 53) reported that interacting with journalists was perceived as a strain or a burden. Few respondents (14%; *n* = 44) reported that they felt that the journalist interactions had been supportive. Instead, the participants strongest agreement seemed to consider the professional behavior of journalists.

There was a weak association between age and positive perception of the interaction with journalists, *r* = 0.122, *p* = 0.031, whereas men and women had rather equal perceptions (*M*_men_ = 2.13, *M*_women_ = 2.08, *t* = 0.55, *p* = 0.58). Similarly, no association was found for educational attainment (*M*_>12yrs_ = 2.12, *M*_≤ 12*yrs*_ = 2.08, *t* = 0.35, *p* = 0.73), or for exposure severity (*M*_high_ = 2.14, *M*_moderate_ = 2.05, *M*_low_ = 1.98, *F* = 0.39, *p* = 0.68). Finally, there was no association between perception of the journalist interaction and posttraumatic stress as assessed at the first survey, *r* = −0.102, *p* = 0.07.

### Perceptions of media exposure

Among the participants who had interacted with journalists and who had been exposed to media coverage of themselves (*n* = 177), the mean score on the eight questions about media exposure was 2.9 (*SD* = 0.75), the median was 3, and the range was 0.25–4.0.

Table 3 provides mean ratings and balance scores for each item on media exposure. Overall, most participants had a neutral view concerning the media coverage or exposure.

**Table 3:** Perceptions of media exposure among 177 tsunami survivors.

**Experience**	**Total**		
	***M***	***SD***	**Balance score**
Support	1.62	1.26	−15.8
Positive	2.47	1.04	36.7
True	3.15	1.02	73.4
Relevant	2.95	1.04	63.8
Strain/burden	1.15	1.17	−46.9
Negative	0.85	1.07	−61.0
Involuntary	0.78	1.25	−63.3
Publicity damage	0.46	0.95	−78.0

*The items range from 0 to 4. The balance score ranges from −100 to +100*.

**Figure 2:**
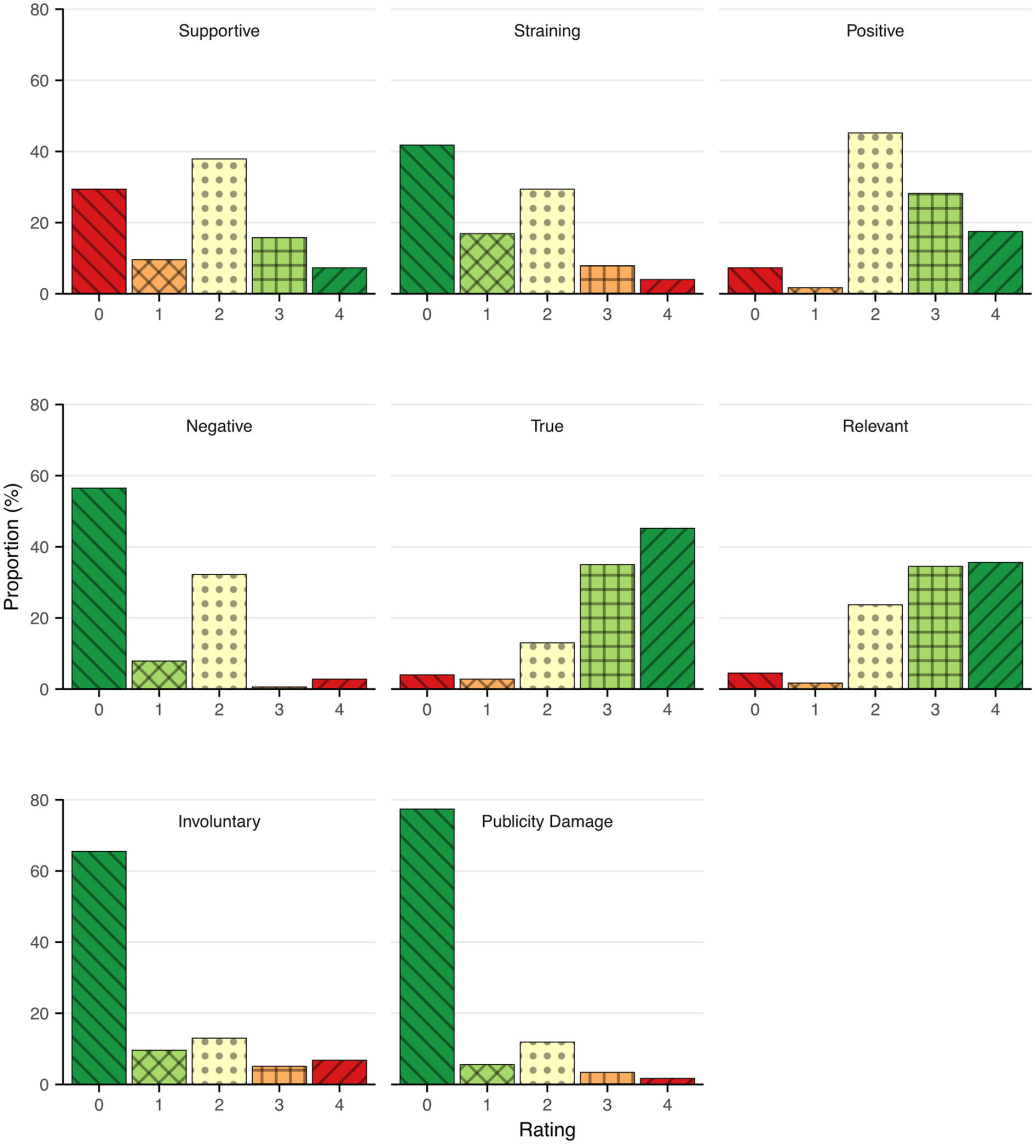
Distribution of Swedish tsunami survivors' perceptions of their media exposure after the disaster. The rating ranges from no agreement (0) to agreeing very much (4).

Figure 2 displays the response patterns to each item. The participants evaluated the media coverage as having been true (80%) and relevant (70%) to a high degree. A few (5%) indicated a sense of being publicity damaged or harmed. The term “publicity damage” is an expression within the Code of Ethics for Press, Radio and Television in Sweden saying “…it is important that the individual is protected from unwarranted suffering as a result of publicity” (19). Thirty-nine percent of the participants indicated that they did not find the media coverage/exposure to be supportive. Twelve percent were involuntary exposed, e.g., was photographed without their knowledge or permission, and ~12% of participants perceived the media exposure to be partly or very much straining. Nine (42%) of the 21 who reported involuntary exposure perceived it as partly or very much straining whereas seven (5.3%) of 133, who disagreed with being involuntary exposed still perceived the exposure as straining.

There was no association between age and positive perception of the media exposure, *r* = 0.14, *p* = 0.068. Men and women had rather equal perceptions (*M*_men_ = 2.99, *M*_women_ = 3.01, *t* = 0.21, *p* = 0.83). Similarly, no association was found for educational attainment (*M*_>12yrs_ = 3.03, *M*_≤ 12*yrs*_ = 3.00, *t* = 0.24, *p* = 0.82) or for exposure severity (*M*_high_ = 2.89, *M*_moderate_ = 2.88, *M*_low_ = 2.88, *F* = 0.009, *p* = 0.99). There was a small association between perception of media exposure and posttraumatic stress at the first survey, *r* = −0.19, *p* = 0.14, indicating that higher scores of posttraumatic stress were associated with a more negative perception of the media exposure. Finally, the association between perceptions of the interaction with journalists was associated with perceptions of the media exposure, *r* = 0.47, *p* < 0.001.

### Trust in media, government, and authorities

The mean score for trust in authorities was 2.34 (*SD* = 0.97), Mdn = 2.25, range = 1–5. The mean score for trust in media was 2.79 (*SD* = 0.92), *Mdn* = 3.0, range = 1–5. The mean trust score was higher for the media than for the authorities, *t* = 10.99, *p* < 0.001. The trust balance score for government, authorities and media (Table 4) indicated a low score for trust in government and governmental authorities as well as evening and local papers, but higher trust for radio, television, and national morning papers.

**Table 4:** Item frequencies and balance scores for trust in government, authorities and media.

**Organization**	**Balance score**	**Trust, %**				
		**Very low**	**Fairly low**	**Neither low nor high**	**Fairly high**	**Very high**
Government	−59.6	49.9	20.4	18.9	9.1	1.6
Government authorities	−48.1	41.9	20.3	23.7	12.3	1.8
Regional authorities	−7.3	22.2	13.4	36.1	24.2	4.1
Municipality	−5.9	22.2	12.1	37.2	24.2	4.3
Radio, TV	13.3	10.7	12.4	40.7	32.3	4.0
National morning papers	11.5	11.8	11.6	41.5	30.6	4.4
Evening papers/tabloids	−40.5	33.9	19.7	33.3	11.2	2.0
Local papers	−14.1	15.8	16.9	48.8	15.9	2.7

*Balance score refers to proportion of low/very low subtracted from the proportion of high/very high, range −100 to +100*.

Finally, we examined whether mean scores on experience of journalist interactions and media exposure were associated with perceptions of trust in authorities and media: participants' experience of the interactions with journalists was associated with their trust in the media, *r* = 0.34, *p* < 0.001, but not with trust in authorities, *r* = 0.06, *p* = 0.39. Participants' experience of media exposure was associated with both trust in media, *r* = 0.47, *p* < 0.001, and with trust in government and authorities, *r* = 0.22, *p* = 0.007.

## Discussion

The aim of this study was to investigate how survivors perceived interactions with journalists and with media exposure and what associations could be identified between these perceptions and institutional trust. The results indicate that the participants, in retrospect, mainly perceived journalists as professional, respectful and supportive and had similar views on being exposed in the media. Their perceptions of the interactions with journalists and the subsequent media exposure were positively associated with their current trust in the media, and their perception of media exposure was also positively associated with their current trust in the government and governmental authorities.

There were 13.5% of the respondents who reported having contact with journalists, and the survivors with the most severe experiences during the tsunami were more likely to report these contacts. In large-scale disasters with thousands of directly exposed survivors, it may be reasonable to expect that only a minority will be in direct contact with the media, whereas disasters with fewer survivors may result in a larger proportion of media contacts within the total number of victims and studied populations (2). Actual disaster exposure varies among survivors, as noted in this study sample, and the higher probability of media contacts among those most severely exposed may reflect various degrees of media interest related to disaster exposure.

Previous studies have pointed out that survivors' opinions of journalists' behaviors can vary widely from good to bad, harmful or supportive, as well as include more neutral and rational reactions (1, 2, 16). Overall, the findings in our study of survivors' perceptions of journalists concur with extant research, although the perceptions in this retrospective study seem to be leaning slightly more toward emotionally neutral statements. It may be that the emotional valence in survivors' recollection of these encounters dissipates in the long term. Nevertheless, in this study, there were still cases of negative perceptions among survivors that might indicate that media ethics and journalists' code of conduct are not applied correctly or alternatively are not adapted sufficiently for disaster media coverage or trauma journalism. A previous study (1) concluded that gatekeepers might be needed between journalists and survivors to prevent ethically dubious conduct. The case for such functions within a crisis organization may be strengthened by the finding that the survivors with the greatest disaster exposure were most likely to report being exposed in the media. This is not surprising, but points to the need for the media to pay attention to ethical journalistic behavior in the context of meeting highly exposed and vulnerable tsunami survivors who may face added challenges and burdens when being interviewed or covered in written and visual media stories.

A majority of the participants perceived the media coverage overall as true, important and relevant, which is in line with previous findings on survivors' perceptions of media coverage and media exposure after traumatic events (1, 2, 14, 15). Nonetheless, 5 percent of the participants agreed to a fairly high degree or very much with statements about the media exposure leading to publicity damage, and 12% perceived the media exposure to have been involuntary. Perceiving one's media exposure to result in publicity damage is an outcome that should be prevented. Importantly, however, although we asked about publicity damage and involuntary media exposure as these aspects were relevant with respect to ethical guidelines, the survey design leaves ample uncertainty regarding how the respondents interpreted these aspects. The findings in this study may serve as a starting point for future investigations into how disaster survivors perceive and experience publicity damage in the context of disaster journalism. Although there might have been changes in media conduct since the tsunami disaster, newer studies may shed light on whether increased survivor participation in the production of the publications (e.g., processes for approving quotes or photos) could decrease the proportion of these negative outcomes.

After large-scale disasters, victims or victim groups may express lack of institutional trust and a sense of being neglected (29, 43). Lack of institutional trust in disaster survivors may raise potential negative implications for health and wellbeing over time (29). At the same time, studies of the 9/11 terrorist attacks in 2001 indicated increased trust in political institutions among a larger population (44). There are also examples of increased trust in different types of institutions (e.g., political, media, justice) after incidents, but the effects can vary across these institutions (45). The question about trust in our study was constructed to facilitate comparisons between the 2004 tsunami disaster survivors and the general Swedish population. The 2005 Swedish national SOM survey on citizens' trust indicated a noticeable increase of trust in Swedish media content between 2004 and 2005. This was especially noticeable regarding national public service radio, local newspapers, national quality press and private satellite channels (39). The same study showed that the generalized trust in Swedish politicians decreased between 2004 and 2005. The tsunami survivors indicated lower trust in authorities than Swedish citizens in general and much lower confidence and trust in the Swedish government in 2005. In addition, the survivors had somewhat lower trust balance scores for radio and TV, but these scores still were higher than those for the national daily press, as compared to the findings from the 2005 SOM survey on the general Swedish population (46). It is to be noted that the tsunami survivors scored their trust in these institutions specifically related to the institutions' handling of the disaster (e.g., crisis management); however, it is equally important to note that both the current survey and the SOM survey asked questions about experiences of trust in 2005, the months and the year following the disaster, a period during which much coverage was devoted to the disaster.

What factors affect survivors' trust in media, government and authorities after a disaster? In general, public trust can be dependent on the content of the media coverage of the disaster. A Swedish study on the public trust in media and authorities after the tsunami disaster 2004 drew the conclusion that when the media were critical of the authorities and the authorities tried to shift the blame or defend themselves, people chose to believe the media reports (18). Additionally, disaster survivors may perceive that the authorities' descriptions of the disaster management are disconnected from their own personal experiences. In this study, there were positive correlations between the survivors' perceptions of interactions with journalists and media exposure and their overall degree of trust in the media, and to some extent, to their degree of trust in the government and governmental authorities. We speculate that the positive association between the survivors' media exposure and their trust in the governmental authorities may be a result of survivors transferring their perceptions of their own media exposure to the media's overall coverage of the disaster. However, in this study we were only able to investigate bivariate, cross-sectional associations, and it is unclear to what extent these associations are confounded by unmeasured variables or methodological constraints. Nonetheless, if personal experiences of the media shape disaster survivors' institutional trust, the media may stand to lose more than they win if they fail to attend to the survivors' needs when prioritizing rapidity in their reporting. The present study suggests that the crisis management of the tsunami created both winners and losers in the court of public opinion.

### Limitations

The findings should be interpreted in light of several limitations. First, the prolonged hiatus between the event and the survey might have brought about a shift in perspectives and evaluations of the early post-disaster events, and there is an unknown degree of recall bias. The cross-sectional design is suboptimal for analyzing associations between events across several years, and a longitudinal study with additional assessments of media perceptions could prevent the threat of confounding in this study. In this regard, it is important to keep in mind that this study investigates survivors' perceptions and experiences in a retrospective fashion. Additionally, non-response and attrition may have negatively affected the representativeness of the sample. We also are fully aware that the study was made in a media context representative of 2004–2005, and we cannot guarantee that we would find the same tendencies and results in a study of the conditions during a media logic in the second or third decade of 2000s. Despite the limitations, this study contributes to a relevant basis for future research on survivors' experiences of media exposure and their trust in media, government and authorities as important parts within the wide context of disaster communication and crisis management following a disaster.

### Implications and future research

The present study focuses on conditions not previously studied. By looking at survivors' experiences from two perspectives, media exposure and trust, we hope to contribute to further research. It could be interesting for future research to explore survivors' views of their trust in media and authorities in longitudinal studies, for example if or how the personal experience from a disaster relates to changes in trust from the immediate to the long-term perspective.

Future studies of survivors from disasters in a more modern media context would be of great interest and importance, not the least in the context of a globalized media situation, multichannel publishing, the frequency of social and digital media use among citizens, authorities and media. How this situation affects survivors' experiences after a disaster is important to follow continuously in future studies.

We suggest that the results of this study can serve as a basis for discussion and knowledge building among the media and authorities. It is also important that crisis support management organizations are informed about survivors' perceptions of media contact. How journalists and media organizations act, meet and cover survivors in the field of disaster and trauma journalism is a topic to which media should pay attention. A crucial point for journalists is awareness and good application of media ethics and codes of conduct. As shown in our study, there are indications of perceived ethical infringements in cases of interviewing and reporting on victims. As the modes of communication and the media logic are rapidly changing (47, 48), we suggest regular updating of guidelines in the field of media ethics, crisis communication, and disaster management.

## Conclusions

**Figure 3:**
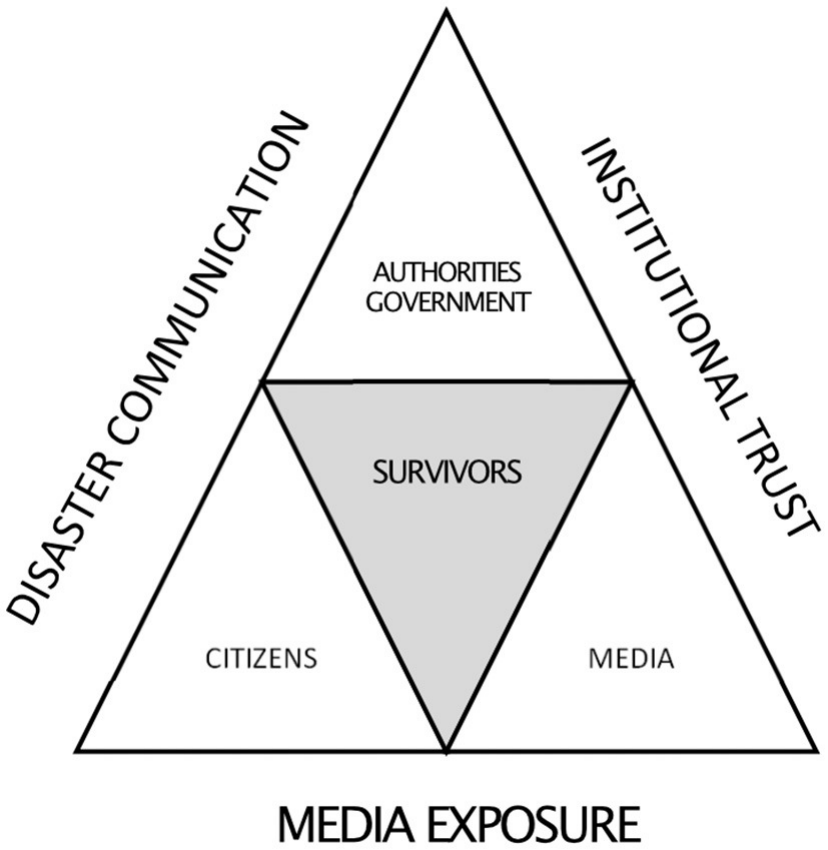
Disaster survivors as interacting actors with media, authorities, government and citizens, in the context of disaster communication, media exposure and institutional trust.

This study clarifies survivors' perceptions of journalists and media in the wake of a disaster and, although this study concerns a disaster several years ago, we hope that it may provide a starting point for studies of more recent events. The survivors generally regarded the journalists' conduct as professional, while for some survivors, the journalists' conduct and subsequent media exposure were associated with negative emotions. Interestingly, it seems as if survivors' perceptions of the journalist interactions are related to their degree of trust in the media and the government and government authorities, whereas their perceptions of media exposure may be related only to their degree of trust in the media. Low institutional trust among survivors and other citizens after a disaster may become a great challenge for authorities and their crisis management and disaster communication. Low trust in the media can also become a democratic problem and an obstacle to effective crisis management in society, underscoring the importance of journalistic responsibility while covering disasters and interviewing survivors. The complex approach in our study shows how all actors with the spotlight on the survivors during a disaster are dependent on the management of disaster communication and media coverage as well as the levels of trust in media and authorities (Figure 3).

## Data availability statement

The original contributions presented in the study are included in the article/Supplementary material, further inquiries can be directed to the corresponding authors.

## Ethics statement

The studies involving human participants were reviewed and approved by the Regional Ethical Vetting Board in Uppsala, Sweden. The patients/participants provided their written informed consent to participate in this study.

## Author contributions

LE had the original idea and constructed the survey questions for the current part of the study. FA and KB conducted the data collection of the full original survey. LE and FA conducted the data curation and formal analysis and wrote the original paper draft. FA conducted the visualization. All authors reviewed, edited, and approved the final version of the manuscript and developed the methods and original idea for the paper.

## Funding

The study was supported by the Swedish National Board of Health and Welfare (Grant Nos. 26356/10-2 and 28337/2011) and the Swedish Civil Contingencies Agency, MSB (Grant No. 2009-6106).

## Conflict of interest

The authors declare that the research was conducted in the absence of any commercial or financial relationships that could be construed as potential conflicts of interest.

## Publisher's note

All claims expressed in this article are solely those of the authors and do not necessarily represent those of their affiliated organizations, or those of the publisher, the editors and the reviewers. Any product that may be evaluated in this article, or claim that may be made by its manufacturer, is not guaranteed or endorsed by the publisher.
