# Supplementary material for: Media perception and trust among disaster survivors: Tsunami survivors' interaction with journalists, media exposure, and associations with trust in media and authorities
Source: Front Public Health. 2022 Aug 2;10:943444. doi: 10.3389/fpubh.2022.943444 (PMC9379093; doi:10.3389/fpubh.2022.943444)
Supplement: Supplementary file 1 [file Data_Sheet_1.docx]

APPENDIX

Reliability Analysis of Perceptions of Journalist Contact and of Perceptions of Media Exposure

Index

[1. Perceptions of Interactions with Journalists 1](#_Toc109139733)

[1.1. Cronbach’s Alpha 2](#_Toc109139734)

[1.2. Inter-Item Correlation and Covariance Matrix 2](#_Toc109139735)

[1.3. Item-Total Statistics 2](#_Toc109139736)

[1.4. Principal Components Analysis 3](#_Toc109139737)

[1.5. Summary 5](#_Toc109139738)

[2. Perceptions of Media Exposure 5](#_Toc109139739)

[2.1. Cronbach’s Alpha 5](#_Toc109139740)

[2.2. Inter-Item Correlation and Covariance Matrix 5](#_Toc109139741)

[2.3. Item-Total Statistics 6](#_Toc109139742)

[2.4. Principal Components Analysis 7](#_Toc109139743)

[2.5. Summary 10](#_Toc109139744)

## Perceptions of Interactions with Journalists

A reliability analysis was conducted with participants who had interacted with journalists and provided responses to all items on the questions about their experiences of their interactions, *n* = 277.

The six questions asked concerned whether the presence of media was perceived as a (1) strain or (2) support, (3) if the journalists showed appropriate consideration for the participant or (4) for others, (5) showed respectful conduct, and (6) behaved professionally.

The participants responded on a five-point scale: “agreeing not at all” (0), “agreeing to a somewhat low degree” (1), “agreeing neither high, nor low/don’t know” (2), “agreeing to a somewhat high degree” (3), “agreeing to a very high degree” (4). The scores for item 1, the question about strain, were reversed before any analysis. A total score was computed by calculating the mean score of all items, and thus the range of the total score is 0 to 4 and a higher score represents a more sympathetic view of journalists on site.

### Cronbach’s Alpha

The internal consistency was high, as the alpha was 0.874 for the six items with their original scoring, and 0.883 if the items were standardized beforehand.

### Inter-Item Correlation and Covariance Matrix

The correlations among individual items ranged from low to high (Table A 1), with the highest correlations between perceptions of the journalists as respectful and professional. There were, overall, lower correlations for the items with higher emotional valence, that is, strain and support.

Table A 1. Inter-Item Correlation and Covariance Matrix

| Experience | 1 | 2 | 3 | 4 | 5 | 6 |
| --- | --- | --- | --- | --- | --- | --- |
| 1. Strain | **1.80** | 0.176 | 0.368 | 0.348 | 0.396 | 0.428 |
| 1. Support | 0.291 | **1.53** | 0.507 | 0.449 | 0.444 | 0.476 |
| 1. Consideration for me | 0.616 | 0.782 | **1.56** | 0.758 | 0.761 | 0.766 |
| 1. Consideration for others | 0.484 | 0.577 | 0.983 | **1.08** | 0.850 | 0.784 |
| 1. Respect | 0.583 | 0.604 | 1.04 | 0.971 | **1.21** | 0.833 |
| 1. Professional behavior | 0.667 | 0.685 | 1.11 | 0.947 | 1.07 | **1.35** |

Note: Correlations in the upper triangle, variances in bold on the diagonal, and covariances in the bottom triangle. The scores for strain are reversed.

### Item-Total Statistics

The following analysis indicates the relationship between each item to the total of the other items (Table A 2). The results indicate that the items with emotional valence had lower associations with the other items.

Table A 2. Item-to-Total Analysis of Journalist Experience

|  | Scale Mean if Item Deleted | Scale Variance if Item Deleted | Corrected Item-Total Correlation | Squared Multiple Correlation | Cronbach's Alpha if Item Deleted |
| --- | --- | --- | --- | --- | --- |
| Strain | 14.63 | 24.28 | .400 | .194 | .903 |
| Support | 14.28 | 23.95 | .486 | .279 | .885 |
| Consideration for me | 13.31 | 20.72 | .798 | .677 | .830 |
| Consideration for others | 13.47 | 22.35 | .807 | .759 | .833 |
| Respect | 13.42 | 21.61 | .835 | .801 | .827 |
| Professional behavior | 13.22 | 21.05 | .839 | .753 | .824 |

### Principal Components Analysis

The items were subjected to a principal components analysis (PCA). The number of components to extract were based on Eigenvalue > 1. As noted in Table A 3, one component was extracted that explained a majority of the variance. As only one component was extracted, no rotations of the solution were assessed. We also analyzed a solution with two components: in this solution, strain was the only item loading meaningfully on the second factor and the results were deemed less satisfactory than the first solution.

Table A 3. Principal Component Analysis, Total Variance Explained

| Component | Initial Eigenvalues | | | Extraction Sums of Squared Loadings | | |
| --- | --- | --- | --- | --- | --- | --- |
|  | Total | % of Variance | Cumulative % | Total | % of Variance | Cumulative % |
| 1 | 3.914 | 65.226 | 65.226 | 3.914 | 65.226 | 65.226 |
| 2 | .837 | 13.954 | 79.180 |  |  |  |
| 3 | .641 | 10.687 | 89.867 |  |  |  |
| 4 | .262 | 4.371 | 94.238 |  |  |  |
| 5 | .210 | 3.496 | 97.735 |  |  |  |
| 6 | .136 | 2.265 | 100.000 |  |  |  |

The Scree plot is provided in Figure A 1 as a visual representation of the extraction criteria, and is provided for clarity.


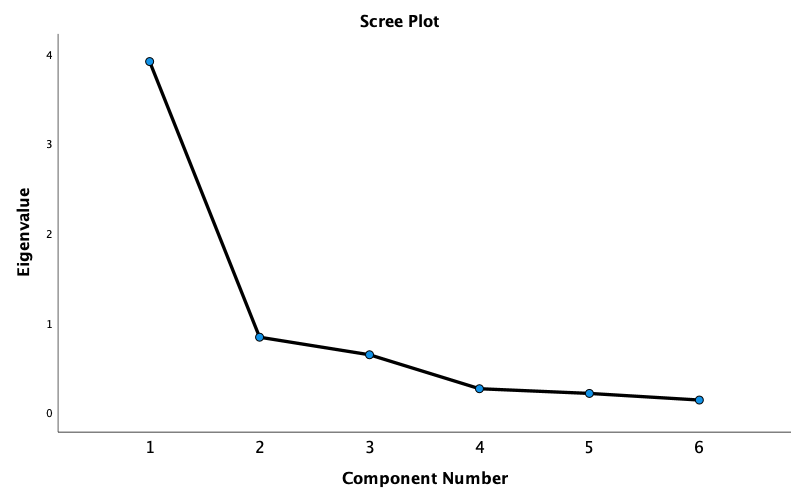


Figure A 1. Scree Plot for Journalist Experience.

Table A 4 shows the communalities at extraction and the factor loadings for the one-component solution. All items showed very high loadings except for the two first items. However, the loadings for these items were still deemed high enough to warrant analyses of these items as one scale.

Table A 4. Communalities at Extraction and the Resulting Factor Loadings for Journalist Experience

|  | Communalities | |  |
| --- | --- | --- | --- |
| Items | Initial | Extraction | Factor Loadings |
| Strain | 1.000 | .265 | .515 |
| Support | 1.000 | .376 | .613 |
| Consideration for me | 1.000 | .784 | .886 |
| Consideration for others | 1.000 | .810 | .900 |
| Respect | 1.000 | .844 | .918 |
| Professional behavior | 1.000 | .834 | .913 |

### Summary

In summary, the analysis indicated that the six items work sufficiently well to be condensed into a meaningful total score represented by the mean score of the items, with the item on strain reversed. The two items that we deem to be of higher emotional valence were slightly less connected to the remaining four items, but the results do not raise any serious objections towards their inclusion. The total score thus represents the survivors’ retrospective recall of interaction experiences with journalists in the direct aftermath of the disaster, including both emotional experiences and perceptions of the journalists' conduct.

## Perceptions of Media Exposure

A reliability analysis was conducted with the 168 participants who had endorsed both interactions with journalists and having been exposed in media, as well as provided responses to all items about their experiences of their media exposure.

The nine questions asked concerned whether participants perceived their own media exposure as (1) supportive, (2) straining, (3) positive, (4) negative, (5) true, (6) relevant, (7) without consent/involuntary, (8) leading to publicity damage, (9) served an important role in providing information.

The participants responded on a five-point scale: “agreeing not at all” (0), “agreeing to a somewhat low degree” (1), “agreeing neither high, nor low/don’t know” (2), “agreeing to a somewhat high degree” (3), “agreeing to a very high degree” (4). The scores for items 2, 4, 7, 8 were reversed before any analysis. A total score was computed by calculating the mean score of all items, and thus the range of the total score is 0 to 4 and a higher score represents a more sympathetic view of media exposure.

### Cronbach’s Alpha

The internal consistency was satisfactory, as the alpha was 0.821 for the nine items with their original scoring, and 0.824 if the items were standardized beforehand. After deletion of item 9 (see below), alpha increased to 0.830.

### Inter-Item Correlation and Covariance Matrix

The correlations among individual items ranged from low to high, with a pattern of higher correlations within the negatively and positively worded items, respectively (Table A 5). The results indicated that item 9 was not meaningfully associated with most other variables except items 1 and 3.

Table A 5. Inter-Item Correlation and Covariance Matrix

| Media Exposure | 1 | 2 | 3 | 4 | 5 | 6 | 7 | 8 | 9 |
| --- | --- | --- | --- | --- | --- | --- | --- | --- | --- |
| 1. Supportive | **1.594** | .194 | .531 | .256 | .316 | .339 | .330 | .158 | .297 |
| 1. Straining | .285 | **1.353** | .270 | .485 | .221 | .204 | .522 | .400 | .156 |
| 1. Positive | .696 | .327 | **1.078** | .528 | .478 | .533 | .414 | .307 | .302 |
| 1. Negative | .348 | .607 | .590 | **1.158** | .467 | .428 | .504 | .372 | .080 |
| 1. True | .414 | .267 | .514 | .522 | **1.077** | .773 | .412 | .279 | .105 |
| 1. Relevant | .449 | .248 | .580 | .483 | .841 | **1.098** | .401 | .221 | .235 |
| 1. Involuntary | .518 | .755 | .534 | .674 | .531 | .523 | **1.545** | .425 | .222 |
| 1. Pub. damage | .187 | .438 | .300 | .376 | .273 | .218 | .496 | **.884** | .129 |
| 1. Important role | .418 | .203 | .349 | .096 | .122 | .274 | .307 | .135 | **1.240** |

Note: Correlations in the upper triangle, variances in bold on the diagonal, and covariances in the bottom triangle. The scores for items 2, 4, 7, 8 are reversed and thus all associations are positive.

### Item-Total Statistics

In the following analysis, the results indicate the relationship between each item to the total of the other items (Table A 6). Here, item 9 again shows a low item-total correlation, and that the internal consistency would be slightly increased if the item was omitted. Similar to the questions about interacting with journalists, the items with clear emotional valence (1, 2, 8) showed lower associations with the other items.

Table A 6. Item-to-Total Analysis of Perceptions of Media Exposure

| Exposure | Scale Mean if Item Deleted | Scale Variance if Item Deleted | Corrected Item-Total Correlation | Squared Multiple Correlation | Cronbach's Alpha if Item Deleted |
| --- | --- | --- | --- | --- | --- |
| Supportive | 24.42 | 32.59 | .460 | .324 | .812 |
| Straining | 23.17 | 33.21 | .467 | .375 | .810 |
| Positive | 23.54 | 31.96 | .663 | .524 | .787 |
| Negative | 22.85 | 32.27 | .605 | .482 | .793 |
| True | 22.88 | 32.78 | .586 | .637 | .796 |
| Relevant | 23.08 | 32.49 | .605 | .647 | .794 |
| Involuntary | 22.79 | 30.60 | .631 | .447 | .789 |
| Pub. damage | 22.48 | 35.09 | .435 | .257 | .812 |
| Important role | 22.95 | 35.78 | .286 | .173 | .830 |

Based on these results, we decided to eliminate item 9. This item, in which the participants rated to what extent they perceived that the media served an important role as a channel for information, seemed to relate poorly to the other questions and tap into aspects not related to their perceptions about being exposed in the media.

### Principal Components Analysis

Items 1-8 were subjected to a principal components analysis. The number of components to extract were based on Eigenvalue > 1. As noted in Table A 7, two components were extracted that together explained 62% of the variance, with the first component alone accounting for 47%.

Table A 7. Principal Component Analysis, Total Variance Explained

| Component | Initial Eigenvalues | | | Sums of Squared Loadings | | | |
| --- | --- | --- | --- | --- | --- | --- | --- |
|  |  | | | Extraction | | | Direct Oblimin |
|  | Total | % of Variance | Cumulative % | Total | % of Variance | Cumulative % | Total |
| 1 | 3.74 | 46.79 | 46.79 | 3.74 | 46.79 | 46.79 | 3.217 |
| 2 | 1.22 | 15.30 | 62.09 | 1.22 | 15.30 | 62.09 | 2.799 |
| 3 | .85 | 10.67 | 72.76 |  |  |  |  |
| 4 | .64 | 8.03 | 80.79 |  |  |  |  |
| 5 | .55 | 6.90 | 87.69 |  |  |  |  |
| 6 | .43 | 5.33 | 93.02 |  |  |  |  |
| 7 | .35 | 4.32 | 97.34 |  |  |  |  |
| 8 | .21 | 2.66 | 100.00 |  |  |  |  |

The Scree plot in Figure A 2 provides a visual representation of the extraction criteria, and is provided for clarity. A principal component analysis was also performed with item 9 included. This resulted in an additional component that included item 9 and to some extent also items 1 and 3, but without a straightforward interpretation (data not shown).

Figure A 2. Scree Plot for Media Exposure.

A direct oblimin rotation of the solution was used to assess the correlation between the components. With this rotation method, the two components were correlated at *r* = 0.424, suggesting against an orthogonal rotation method. The sums of squared loadings after rotation are included in the rightmost column in Table A 7.

Table A 8 shows the communalities at extraction and the unrotated and rotated factor loadings for the two-component solution. The component separation was not very well supported in that most items loaded on both components and, as noted, the correlation between components was rather high. Importantly, the main difference between the extracted components seemed to relate to whether the item was positively or negatively worded.

Table A 8. Communalities at Extraction and the Resulting Factor Loadings for Journalist Experience

|  | Communalities | Component Matrix | | Rotated Matrix | |
| --- | --- | --- | --- | --- | --- |
| Items | Extraction | 1 | 2 | 1 | 2 |
| Supportive | .399 | .556 | -.300 | .632 | .256 |
| Straining | .704 | .583 | .604 | .243 | .831 |
| Positive | .633 | .757 | -.244 | .785 | .449 |
| Negative | .601 | .754 | .183 | .587 | .708 |
| True | .707 | .746 | -.387 | .841 | .354 |
| Relevant | .755 | .740 | -.456 | .866 | .306 |
| Involuntary | .634 | .736 | .303 | .517 | .768 |
| Pub damage | .534 | .554 | .477 | .274 | .730 |
| Important role | .399 | .556 | -.300 | .632 | .256 |

### Summary

The analyses of the questions about perceptions of media exposure indicated that eight of the original nine items work sufficiently well to be condensed into one construct. Alternatively, the division of questions into two constructs may be of relevance particularly in psychometric investigations into negative and positive perceptions of media exposure, respectively.

A total score represented by the mean score of the items, with the negatively worded items reversed, is seen as meaningful. The total score thus represents the survivors’ retrospective recall of experiences of having been exposed in the media after interacting with journalists in the aftermath of the disaster.
